# Simultaneous Detection of Antigen-Specific IgG- and IgM-Secreting Cells with a B Cell Fluorospot Assay

**DOI:** 10.3390/cells1010015

**Published:** 2012-03-21

**Authors:** Mattia Bonsignori, M. Anthony Moody

**Affiliations:** Duke Human Vaccine Institute, Duke University Medical Center, 2 Genome Court, Durham, NC 27710, USA; E-Mail: tony.moody@duke.edu

**Keywords:** fluorospot assay, antibody-secreting cells, B cells, multiplex, quantum-dot nanocrystals

## Abstract

The traditional enzyme-linked immunospot (ELISpot) assay is the gold standard for the enumeration of antigen-specific B cells. Since B cell availability from biological samples is often limited, either because of sample size/volume or the need of performing multiple analyses on the same sample, the implementation of ELISpot assay formats that allow the simultaneous detection of multiple antibody types is desirable. While dual-color ELISpot assays have been described, technical complexities have so far prevented their wide utilization as well as further expansion of their multicolor capability. An attractive solution is to replace the chromogenic reaction of the traditional ELISpot assay with a fluorescent detection system (fluorospot assay). Fluorospot assays using fluorophore-conjugated secondary antibodies in conjunction with fluorescence enhancers, FITC/anti-FITC and biotin/avidin amplification systems and dedicated equipment for spot detection have been developed to enumerate T-cells secreting two or three specific cytokines and, more recently, IgG and IgA antibody-secreting cells (ASCs). We hereby report a method for a multiplex B cell fluorospot assay that utilizes quantum-dot nanocrystals as reporters without further amplification systems or need of dedicated equipment. With this method we simultaneously enumerated HIV-1 gp41 envelope glycoprotein-specific IgG and IgM antibody-secreting cells with sensitivity comparable to that of the traditional ELISpot assay.

## 1. Introduction

The gold standard for the enumeration of immune cells secreting a specific protein, either antibodies or cytokines, is the enzymed-linked immunospot (ELISpot) assay. In the ELISpot assay, cells are cultured for sufficient time to allow secretion of the protein of interest onto wells with a porous membrane surface appropriately coated with an antigen. The membrane retains the secreted protein of the desired specificity in the location where each cell was sitting. After removing the cells, their footprints are visualized as discrete spots by the means of a colorimetric reaction [1,2]. The ELISpot assay is widely used to enumerate antibodies secreted by B cells [3,4,5] and cytokines secreted by T cells [6,7,8]. A limitation of the traditional ELISpot assay is that it can only detect a single protein of interest [9,10,11,12]. The ability to detect multiple antibody specificities and/or isotypes on the same specimen is not only desirable but is critical when the specimen availability is limited. 

Modifications of the original ELISpot technique have been described to allow the simultaneous detection of two proteins of interest: by using a combination of two chromogens and two detecting antibodies tagged with different proteins, dual-color ELISpot assays have been implemented to concurrently detect two antibody isotypes secreted by B cells [13,14,15] or two cytokines secreted by T cells [16,17,18,19,20,21,22]. Albeit dual-color ELISpot assays have been successfully utilized [19,22], interferences between enzymatic reactions and loss in resolution have been reported, and the overall technical complexity of performing and analyzing the data generated with dual-color ELISpot assays hindered its wide utilization [11,12,20].

More recently, the traditional ELISpot assay has been further modified by replacing the chromogenic reaction with fluorophore-conjugated antibodies (fluorospot assay) for the simultaneous detection of two [9,10,23] and three [12] proteins secreted by T cells. Even more recently, a commercial kit for the simultaneous detection of IgG and IgA antibody-secreting cells (ASCs) with a B cell fluorospot assay has been released.

As novel uses of the flurospot assay and exciting implementations of detection systems and data analysis are being developed, we hereby describe a modified method for the B cell fluorospot assay in which fluorophores are replaced with quantum-dot nanocrystals to simultaneously detect antigen-specific IgG and IgM ASCs.

## 2. Results and Discussion

### 2.1. Comparison of the Sensitivity and Linear Range of Detection of the B cell Fluorospot Assay and the Traditional ELISpot Assay in Enumerating IgG and IgM ASCs

We first evaluated the sensitivity of the B cell fluorospot assay in a single-color format using the traditional B cell ELISpot assay as control. IgG-7B2 and IgM-2B9 cells-two immortalized B cell hybridoma cell lines secreting anti-HIV-1 gp41 Env IgG and IgM mAbs, respectively-were plated at cell densities ranging from 100 cells/well to 5000 cells/well and ASCs were detected by using anti-IgG and anti-IgM mAbs conjugated to either horseradish peroxidase for the traditional ELISpot or quantum dot nanocrystals emitting at wavelengths of 605/40 nm and 655/40 nm, respectively, for the B cell fluorospot assay. 

Using the B cell fluorospot assay, we enumerated 50 ± 11 (mean ± s.d.) IgG-7B2 ASCs at 100 cells/well, 222 ± 18 ASCs at 500 cells/well, 417 ± 12 ASCs at 1000 cells/well and 1721 ± 64 at 5000 cells/well (Table 1A). The ratio of spots per plated IgG-7B2 cells (spot:cell ratio) ranged from 0.50 at the lowest cell density to 0.34 at 5000 cells/well and linear regression analysis demonstrated that ASC detection was within the linear range of the assay (r = 0.999, *p* < 0.001) (Table 1A). The traditional ELISpot assay detected comparable numbers of IgG ASCs: 52 ± 9 ASCs at 100 cells/well, 232 ± 19 ASCs at 500 IgG-7B2 cells/well, 416 ± 18 ASCs at 1000 IgG-7B2 cells/well and 1669 ± 260 ASCs at 5000 cells/well (Table 1A) with a similar spot:cell ratio (from 0.52 to 0.33). Also for the traditional ELISpot assay, linear regression analysis demonstrated that ASC detection was in the linear range (r = 0.998, *p* < 0.001) (Table 1A).

Table 1Linearity of detection and comparison between numbers of (**A**) IgG and (**B**) IgM ASCs/well obtained with the traditional ELISpot and the novel B cell fluorospot assays.

**(A):** 

cells/well	7B2-IgG			
	ELISpot		Fluorospot	
	ASC/well^a^	spot:cell ratio	ASC/well ^a^	spot:cell ratio
0	0	n/a	0	n/a
100	52 ± 9	0.52	50 ± 11	0.50
500	232 ± 19	0.46	222 ± 18	0.44
1,000	416 ± 18	0.42	417 ± 12	0.42
5,000	1,669 ± 260	0.33	1,721 ± 64	0.34
	**Linearity of the range of detection**			
*r*	0.99<0.0001		0.998<0.0001	
*p*

**(B):** 

cells/well	2B9-IgM			
	ELISpot		Fluorospot	
	ASC/well ^a^	spot:cell ratio	ASC/well ^a^	spot:cell ratio
0	0	n/a	0	n/a
100	9 ± 1	0.09	17 ± 0	0.17
500	52 ± 2	0.10	67 ± 1	0.13
1,000	95 ± 4	0.10	138 ± 8	0.14
5,000	453 ± 11	0.09	518 ± 22	0.10
	**Linearity of the range of detection**			
***r***	0.999<0.0001		0.997<0.0001	
***p***				

**Figure 1:**
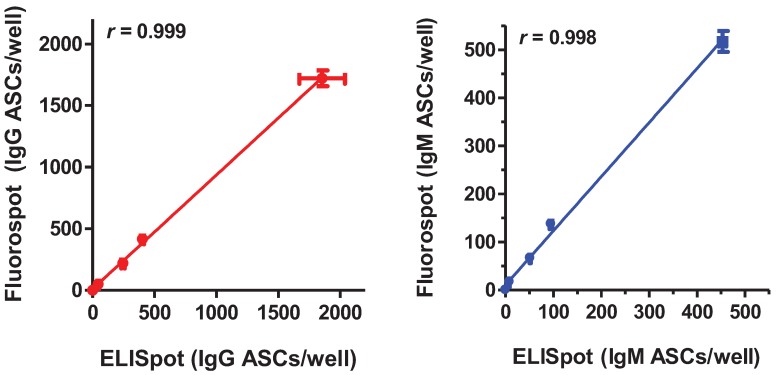
Correlation between antibody-secreting cell counts obtained with the traditional ELISpot and the novel B cell fluorospot assay. IgG ASCs (on the left) and IgM ASCs (on the right) counted with the ELISpot assay are plotted on the *x*-axis and those counted with the fluorospot assay in side-by-side experiments are plotted on the *y*-axis.

The counts of IgM-2B9 ASCs obtained with the two techniques were also comparable. The B cell fluorospot assay enumerated 17 ± 0 IgM ASCs at 100 cells/well, 67 ± 1 ASCs at 500 cells/well, 138 ± 8 ASCs at 1000 cells/well and 518 ± 22 ASCs at 5000 cells/well (Table 1B) while, with the traditional ELISpot assay, we obtained 9 ± 1, 52 ± 2, 95 ± 4 and 453 ± 11 ASCs at 100, 500, 1000 and 5000 cells/well, respectively (Table 1B). The spot:cell ratio ranged from 0.10 to 0.17 in the fluorospot assay and from 0.09 to 0.1 in the traditional ELISpot assay. Linear regression analysis demonstrated that both assays were within their linear range of detection (r = 0.999, *p* = 0.0005 for the fluorospot assay and r = 0.999, *p* = 0.0014 for the ELISpot assay) (Table 1B). The fluorospot assay consistently returned slightly higher counts of IgM ASCs than the traditional ELISpot assay but the difference was not statistically significant (*p* = 0.1, paired Student-t test). No ASCs were detected in any assay in absence of cells (Table 1A,B) and no spots were visible when wells were observed using the optical filter for the mismatched wavelength (data not shown). Overall, a linear relationship was observed between results obtained with the B cell fluorospot assay and the traditional ELISpot assay for both IgG ASCs (r = 0.999; *p* < 0.0001) and IgM ASCs (r = 0.998; *p* < 0.0001) (Figure 1).

Taken together, these data demonstrate that this format of B cell fluorospot assay is as sensitive as the traditional ELISpot assay in detecting isotype-specific ASCs.

### 2.2. Multiplex Detection of IgG and IgM Antibody-Secreting Cells

**Figure 2:**
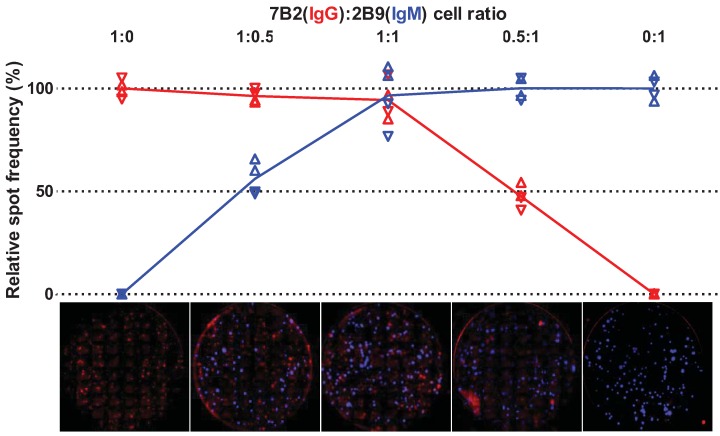
Simultaneous detection of IgG and IgM antibody-secreting cells with a multiplex B cell fluorospot. IgG-7B2 and IgM-2B9 cells were mixed at various ratios (1:0, 1:0.5 and 1:1, as described in the text) and incubated in anti-IgG+M-coated wells. The plot shows the IgG (in red) and IgM (in blue) ASC frequency in each of duplicated wells of two experiments (▼ and ▲) relative to the number of spots counted in wells in which only one cell line was cultured. Dotted lines show the expected values at different cell ratios. Representative wells are shown.

Next, we used the B cell fluorospot assay to enumerate IgG and IgM ASCs simultaneously. IgG-7B2 and IgM-2B9 cells were mixed at various ratios (including cultures with only one cell line), incubated in wells coated with both anti-IgG and anti-IgM mAbs and ASCs were detected using both anti-IgG-Qdot_605_ and anti-IgM-Qdot_655_ mAbs. Experimental conditions and results of two independent experiments with duplicate wells for each condition are summarized in Table 2. Results were also expressed as the frequency of IgG and IgM ASCs relative to the number of ASCs counted from cultures containing only one cell line (Figure 2). 

**Table 2:** IgG and IgM antibody-secreting cells enumerated in two independent experiments using the multiplexed B cell fluorospot assay.

Condition	cells/well			IgG ASCs				IgM ASCs		
	IgG-7B2	IgM-2B9		Well 1	Well 2	Average		Well 1	Well 2	Average
**Experiment** **1**										
1:0	1,000	0		955	987	971		0	0	0
1:0.5	1,000	500		915	906	911		43	47	45
1:1	1,000	1,000		827	940	884		79	77	78
0.5:1	500	1,000		465	527	496		69	75	72
0:1	0	1,000		0	0	0		76	67	72
**Experiment 2**										
1:0	500	0		360	325	343		0	0	0
1:0.5	500	1,000		343	334	339		94	92	93
1:1	500	2,000		304	365	335		175	145	160
0.5:1	250	2,000		161	140	151		178	198	188
0:1	0	2,000		0	0	0		195	183	189

The frequencies of IgG and IgM ASCs detected in cultures containing IgG-7B2 and IgM-2B9 cells at the highest density were, respectively, 94.3% and 96.5% of ASCs from cultures with a single cell type (Figure 2). When IgG-7B2 or IgM-2B9 were co-cultured using only half of the inoculum for one cell line (“1:0.5” and “0.5:1” in Table 2), the frequencies of observed IgG and IgM ASCs consistently reflected the variations in the amount of input cells in each well: 47.5% of IgG ASCs and 56.1% of IgM ASCs (Figure 2). Overall, the frequencies of IgG and IgM ASCs at each combination of cell densities were within 3.0% and 2.4% of the expected ASC counts, respectively, both of which were well less than one standard deviation from the expected value.

These results demonstrate that the multiplex B cell fluorospot retains the sensitivity of the single-color format and that even a minority of the total ASCs in culture can be successfully discriminated. No spots were detected in our control wells with only one cell line when the mismatched emission filter was used, indicating absence of cross-reactivity or bleed over of the detecting mAbs. 

### 2.3. Multiplex Detection of HIV-1 gp41 Env-Specific ASCs

**Figure 3:**
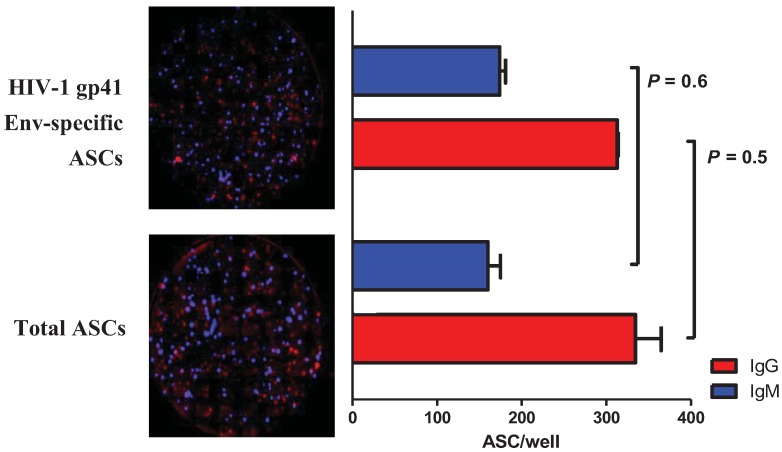
Muliplex detection of total or HIV-1 gp41 Env-specific IgG and IgM antibody-secreting cells. IgG-7B2 and IgM-2B9 cells were co-cultured using either HIV-1 MN gp41 Env (top picture) or a mixture of anti-human IgG and IgM mAbs (bottom picture) as capture reagents to detect IgG-secreting (in red) and IgM-secreting (in blue) ASCs. The graph on the right shows no statistically significant difference in the numbers of ASC/well detected using the two B cell fluorospot assay formats (n = 2).

To investigate if the multiplex B cell fluorospot assay maintained the same level of sensitivity when used to identify antigen-specific ASCs, we tested a mixture of IgG-7B2 and IgM-2B9 cells in wells coated with either a mixture of anti-IgG and anti-IgM mAbs or recombinant HIV-1 gp41 envelope glycoprotein (Env). Since both IgG-7B2 and IgM-2B9 cells secrete anti-gp41 Env antibodies, no differences in total and gp41 Env-specific ASC counts were expected. Indeed, results from two independent experiments showed no statistically significant difference in the counts of gp41 Env-specific *vs.* total IgG ASCs (313 ± 13 *vs.* 335 ± 4 ASCs; *p* = 0.55, Student’s t-test) and total IgM ASCs (174 ± 9.9 *vs.* 160 ± 21.2 ASCs; *p* = 0.49, Student’s t-test) (Figure 3). 

These data demonstrate that this B cell fluorospot format allows the simultaneous enumeration of antigen-specific ASCs of two isotypes.

### 2.4. Discussion

The ELISpot assay is widely used and represents the gold standard for enumeration of antigen-specific antibody-secreting cells and T cells producing specific cytokines. The fluorospot assay is a modification of the traditional ELISpot assay in which the chromogenic reaction used to visualize discrete spots is replaced with a fluorescent detecting system. Such modification has the advantage to detect multiple secreted proteins simultaneously without reducing the sensitivity of the system. 

In this report we described a method to perform a B cell fluorospot assay that uses: anti-human IgG and anti-human IgM detecting mAbs conjugated with quantum dot nanocrystals for the detection of ASCs without further signal amplification systems; a non-dedicated fluorescent microscope equipped with quantum-dot-specific filters for acquiring images, and the ImageJ software for counting spots. Using a dual-color format, we visualized and enumerated simultaneously HIV-1 gp41 envelope glycoprotein-specific immortalized human memory B cells secreting IgG and IgM monoclonal antibodies. The counts of IgG and IgM fluorescent spots detected in the isotype-specific (total IgG/IgM) and the antigen-specific formats of the assay were comparable and strongly correlated with results obtained using the traditional ELISpot assay. Moreover, we demonstrated that in the dual-color format a minority of ASCs from a mixed cell population could be accurately enumerated.

Methods to perform fluorospot assays have been proposed and used for almost a decade to simultaneously detect two or three cytokines secreted by T cells [10,11,12,23] and recently a kit for the multiplexed detection of IgG and IgA antigen-specific ASCs has been commercialized. The B cell fluorospot assay in a single-color format has been also recently adapted to quantify the antibody secreting from individual plasma cells [9]. Multiplexing of the fluorospot assay has been achieved through various ingenious methods: dual-color fluorospot assays use fluorophore-conjugated secondary antibodies in conjunction with fluorescence enhancers, a FITC/anti-FITC and biotin/avidin amplification systems and use of dedicated equipment for spot detection and enumeration. A three-color fluorospot assay for cytokine detection has been described that uses fluorophore-labeled species-specific anti-Ig antibodies [12]. However, unavailability of additional amplification systems compatible with FITC/anti-FITC and biotin/avidin reactions and the limited amount of species from which highly functional antibody pairs are derived may pose limitations to further increase the multi-color functionality of this technology [11].

In the attempt to develop a B cell fluorospot assay using non-dedicated equipment and to identify a format that could readily be scaled up to more than three colors, we had initially tried to use fluorophore-conjugated detecting antibodies. This approach did not succeed for at least three reasons: photo-bleaching prevented a sustained detectable signal throughout multiple and prolonged exposures of the same field; the intensity of the signal obtained from the spots was too low to be consistently distinguished from background (we did not use any fluorescence enhancing compound); and overlaps of emission spectra that could not be compensated in a system in which the final readout relays on a visual analysis at the microscope introduced substantial artifacts. To address these issues, we replaced fluorochromes with quantum-dot nanocrystals as reporters. The main advantages that we found were that quantum-dot nanocrystals did not photo-bleach even after multiple exposures of each field for several seconds and were not as photosensitive as fluorophores, which allowed easier manipulation of the plates; their narrow emission peaks, in conjunction with specific filters, resulted in clear signals in the corresponding channel without bleed over into other channels-a pivotal characteristic to allow further multicolor capabilities-and finally, a higher level of brightness allowed clear detection of spots with only minimal and simple linear adjustment of the color levels.

## 3. Experimental Section

### 3.1. Cell Lines

Two immortalized human B cell hybridoma cell lines, IgG-7B2 (a gift of James Robinson, Tulane University, New Orleans, LA, USA) and IgM-2B9 (generated by Kwan-Ki Hwang, Duke Human Vaccine Institute, Durham, NC, USA) were used for this study. Both cell lines secrete monoclonal antibodies directed against the HIV-1 gp41 envelope glycoprotein.

### 3.2. Conjugation of Anti-Human IgG and IgM to Quantum-Dot Nanocrystals

Mouse anti-human IgG Fc (HP6043P, Hybridoma Research Laboratory; Baltimore, MD, USA) and mouse anti-human IgM Fc (HP6083P, Hybridoma Research Laboratory) were conjugated to quantum-dot nanocrystals emitting at wavelengths of 605/40 nm and 655/40 nm, respectively, using Qdot antibody conjugation kits (Q22001MP and Q22021MP; Invitrogen; Carlsbad, CA) and following manufacturer’s instructions. Briefly, quantum-dot nanocrystals were activated by mixing 125 µL Qdots with SMCC followed by a 1-hour incubation at RT. Purified antibodies were reduced by incubating 300 µL of 1 mg/mL solution in PBS with 6.1 µL DTT for 30 min. Both activated Qdots and reduced antibodies were desalted by using the columns provided with the kit and collected in a final volume of 500 µL/each. Purified antibodies were conjugated to the respective Qdots by co-incubating them for 1 h at RT. The conjugation reaction was quenched by adding 10 µL of 10 mM solution of 2-mercaptoethanol and incubating for 30 min at RT. Each sample was concentrated to 40 µL by centrifugation at 4,000 × *g* in the provided ultrafiltration devices and conjugated antibodies were retrieved by column separation as per manufacturer instructions. Concentration of the collected conjugates was determined by repeated measurements of the optical density using a Nanodrop 1000 spectrophotometer (Thermo Scientific; Wilmington, DE, USA).

### 3.3. B Cell Fluorospot Assay

Ninty-six-well black wall, clear bottom plates (3603, Corning; Corning, NY, USA) were coated with 100 µL/well of mouse anti-human IgG Fd (HP6046P, Hybridoma Research Laboratory) and/or mouse anti-human IgM Fc (HP6081P, Hybridoma Research Laboratory) at 10 µg/mL overnight at 4 °C. For antigen-specific B cell fluorospot assays, plates were coated with 10 µg/mL recombinant HIV-1 MN gp41 envelope glycoprotein. Plates were manually washed 2X with PBS and blocked with 200 µL/well of PBS/1% BSA for 1 h at 37 °C. Meanwhile, IgG-7B2 cells and/or IgM-2B9 cells were collected, washed twice by centrifuging at 1500 rpm for 5 min at 22 °C and resuspended in RPMI 1640 containing 10% FCS supplemented with glutamic acid and penicillin/streptomycin. Viability was checked by trypan blue exclusion. Cells were plated in 100 µL/well at the desired concentration and incubated for 3 h at 37 °C, 5% CO_2_ in humidified atmosphere. Plates were then washed 8 times with PBS/1% BSA/0.05% Tween-20. Fifty µL/well of detecting antibodies were added at the optimized concentrations of 20 nM for the anti-IgG-Qdot_605_ mAb and 40 nM for the anti-IgM-Qdot_655_ mAb. In some preliminary experiments in a single-color format we also used a commercially available goat anti-human IgG conjugated with Qdot_605_ (Q11201MP, Invitrogen). After a 1-hour incubation at RT in the dark, plates were manually washed 4 times with PBS/0.01% Tween-20 and 4 times with PBS. Plates were gently tapped in-between washes to facilitate cell detachment. Finally, test wells were filled with 100 µL/well PBS and multiple blank wells in each row and column of the plate were filled with one drop of polystyrene microparticles (CompBeads Negative Control, 552,843, Beckton Dickinson; Franklin Lakes, NJ, USA) to allow easy focusing during image acquisition at the microscope.

### 3.4. Acquisition of Fluorescent Images

Images were acquired using a Nikon Eclipse TE2000-E fluorescent microscope equipped with a Chroma Technology 470DCXR-BS dichroic beam splitter (Chroma Technology; Bellows Falls, VT, USA), 605/40 nm (D605/40 m; Chroma Technology) and 655/20 nm (D655/20; Chroma Technology) emission filters and NIS Element AR microscope imaging software (Nikon Instruments; Melville, NY, USA). Images were acquired at a 10× magnification, 2 × 2 bidding and optimized exposure times of 1 to 3 s. A linear adjustment of the color levels was preliminary determined and fixed for images taken at each wavelength and routinely applied to every digital image. No gamma correction or non-linear adjustments, digital filters, cropping or any adjustment limited to partial areas of any image were applied [24]. For each well, we took 81 overlapping images (covering the whole well surface) for each wavelength tested (including a bright light field). Images from each well were stitched using the function built in the software and converted in tagged image file format (TIFF). Stage movements and focusing were automated at single well level using the imaging software while filter cube changing and acquisition of multiple wells were managed by the operator. Spots visualized in each channel were manually counted from the composite images of each well using the ImageJ software [25,26].

### 3.5. Traditional ELISpot Assay

We performed the traditional ELISpot assay as previously described [5]. Briefly, 96-well Immobilon P flat bottom plates (Millipore; Billerica, MA), pre-treated with 70% ethanol for 30 s and washed twice with PBS, were coated overnight at 4 °C with 100 µL of mouse anti-human IgG Fd (HP6046P, Hybridoma Research Laboratory) and/or mouse anti-human IgM Fc (HP6081P, Hybridoma Research Laboratory). Plates were washed twice with PBS and blocked for 2 h at 37 °C with PBS/1% BSA. Cells were incubated for 3 h at 37 °C, 5% CO_2_ in humidified atmosphere. Plates were then washed 4 times with PBS/0.05% Tween-20 using an ELx405 automated plate washer (BioTek Instruments; Winooski, VT, USA) and 100 µL of a 1:4000 dilution of mouse HRP-conjugated anti-human IgG or IgM mAbs (HP6043HRP and HP6083HRP, Hybridoma Reagent Laboratory) were added for 1 h at RT. Plates were washed twice with PBS/0.05% Tween-20 and twice with PBS. Spots were developed adding 100 µL/well of 3-amino-9-ethylcarbazole at 0.3 mg/mL diluted in 0.1 M sodium acetate pH 5.0 and 0.03% hydrogen peroxide for 5 min in the dark. After extensive washing of the membranes with distilled water and overnight drying, spots were counted with the ImmunoSpot Series 3B Analyzer and ImmunoSpot 4.0 software (CTL, Cleveland, OH, USA).

## 4. Conclusions

The work presented describes a method to perform a B cell fluorospot assay for the multiplexed detection of antibody-secreting cells using quantum dot nanocrystals as reporters.
